# A systematic review of reviews on the advantages of mHealth utilization in mental health services: A viable option for large populations in low-resource settings

**DOI:** 10.1017/gmh.2024.39

**Published:** 2024-04-04

**Authors:** Mohsen Khosravi, Ghazaleh Azar

**Affiliations:** 1 Department of Healthcare Management, School of Management and Medical Informatics, Shiraz University of Medical Sciences, Shiraz, Iran; 2 Department of Consultation and Mental Health, Yasuj University of Medical Sciences, Yasuj, Iran

**Keywords:** digital health, telehealth, mHealth, mental health, patient-centered care, healthcare quality, health services, delivery of health care

## Abstract

Global mental health services face challenges such as stigma and a shortage of trained professionals, particularly in low- and middle-income countries, which hinder access to high-quality care. Mobile health interventions, commonly referred to as mHealth, have shown to have the capacity to confront and solve most of the challenges within mental health services. This paper conducted a comprehensive investigation in 2024 to identify all review studies published between 2000 and 2024 that investigate the advantages of mHealth in mental health services. The databases searched included PubMed, Scopus, Cochrane and ProQuest. The quality of the final papers was assessed and a thematic analysis was performed to categorize the obtained data. 11 papers were selected as final studies. The final studies were considered to be of good quality. The risk of bias within the final studies was shown to be in a convincing level. The main advantages of mHealth interventions were categorized into four major themes: ‘accessibility, convenience and adaptability’, ‘patient-centeredness’, ‘data insights’ and ‘efficiency and effectiveness’. The findings of the study suggested that mHealth interventions can be a viable and promising option for delivering mental health services to large and diverse populations, particularly in vulnerable groups and low-resource settings.

## Graphical abstract

**Figure:**
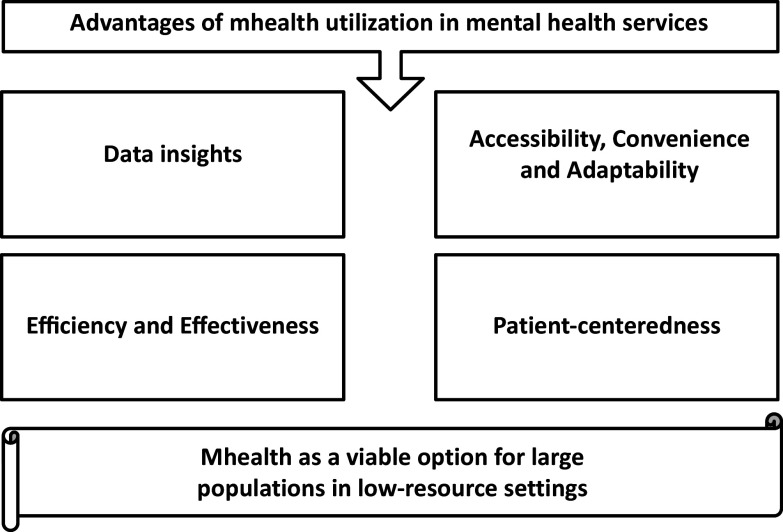

## Impact statement

The findings of the study can be utilized by future researchers, policymakers and healthcare administrators from diverse socioeconomic and geographical backgrounds providing them a more detailed insight regarding the advantages of utilizing mHealth in mental health services in diverse settings.

## Introduction

Global mental health services are facing numerous challenges that hinder access to high-quality care (Collins et al., 2013;Saxena, 2016; Wainberg et al., 2017). These challenges include stigma, which can prevent people from seeking the care they need, and a shortage of trained mental health professionals, particularly in low- and middle-income countries (Saxena, 2016; Wainberg et al., 2017).

Mental health is a state of well-being that encompasses the biological, psychological and social aspects of an individual’s life. It can be characterized by the absence of mental illness and the presence of positive mental states (Manwell et al., 2015).

Mental health services are often fragmented and poorly integrated with other health services, making it difficult for people to access the care they need (Wainberg et al., 2017). There is also a lack of research capacity for implementation and policy change, which contributes to the current mental health treatment gap (Wainberg et al., 2017). Many people who need mental health care do not have access to evidence-based interventions, particularly in low-resource community or primary care settings (Wainberg et al., 2017). There is a need for innovations and global exchange of information, evidence and knowledge to address the diverse mental health needs of different populations (Saxena, 2016).

In settings with limited resources, populations, particularly adolescents, encounter challenges in accessing mental health services. These challenges stem from a scarcity of resources, an absence of standardized tools for monitoring service quality, frail health systems and the complexities of diverse cultural environments (Lund, 2018; Galagali and Brooks, 2020). Furthermore, the challenges encompass the absence of governmental prioritization of mental health, extensive distances to reach treatment facilities, inadequate availability of trained personnel and the prevailing perception that mental illness is non-life-threatening, resulting in a diminished emphasis on mental health services (Mekonen et al., 2022).

Mobile health, or mHealth, is the utilization of mobile devices like smartphones and tablets in healthcare. It includes applications for accessing medical information, monitoring health, tracking behaviors such as diet and exercise and facilitating patient-provider communication (Maaß et al., 2022).

mHealth, has the capacity to serve as a primary mode of mental health care, as well as augment treatment and provide education (Kruse et al., 2022). The widespread adoption and enhanced capabilities of mobile devices have significant implications for the provision of mental health services. The effective utilization of mobile applications can increase access to evidence-based care, empower consumers with information, promote active engagement in treatment, facilitate the implementation of evidence-based practices and improve posttreatment care (Price et al., 2014).

Due to the novelty of the topic and the fact that mHealth has only recently been utilized within the healthcare service delivery systems, there seems to be a growing need to understand the effects of such novel platform and particularly its advantages in diverse aspects of service delivery network. According to the authors’ observations, there had been only a few studies that review the advantages of mHealth applications on mental health services due to the novelty of the topic (Berrouiguet et al., 2016; Kruse et al., 2022). A systematic review highlighted the advantages of mHealth text messaging, including enhanced ecological momentary assessment, improved patient engagement and better mental health outcomes for individuals with chronic conditions. However, it also discussed potential obstacles and limitations in clinical settings, such as patient noncompliance, risk of social isolation and the necessity for a deeper comprehension of patient behaviors and technological progress (Berrouiguet et al., 2016). Another systematic review indicated that mHealth interventions have the potential to offer education, enhance treatment and function as the principal method in mental health care (Kruse et al., 2022).

Current study aimed to be a pioneer in conducting a systematic review of reviews on the existing literature in this context. The goal was to provide accumulated evidence-based data regarding the advantages of mHealth utilization in different aspects of the service delivery network. This information could later be utilized by future researchers, policymakers and healthcare administrators providing them a more detailed insight regarding the topic.

## Materials and methods

This paper, employing a qualitative approach conducted in 2024. The question of the research was designed as: “what are the advantages of utilizing mHealth in mental health services?” A thematic analysis was performed on the data acquired through a Systematic Review that conducted a search within multiple databases following the Preferred Reporting Items for Systematic Reviews and Meta-Analyses (PRISMA) guidelines (Page et al., 2021). The PRISMA 2020 guidelines followed by the present study to guarantee the rigor and reliability of the research can be found in Supplementary Appendix 3 (PRISMA 2020 checklist).

### Data collection and search method

As presented in Table 1, a comprehensive investigation was carried out to locate all review articles published between 2000 and 2024 that discuss the advantages of mHealth in mental health services. The databases utilized for this search included PubMed, Scopus, Cochrane and ProQuest. MeSH terms were employed to classify all keywords into three categories: effects, mHealth and mental health. During the process, we employed more comprehensive keywords, such as “effects” or “outcome”, to explore and incorporate those studies that had mentioned the benefits of using mHealth in certain sections of their results, even though their primary objective was not exclusively to investigate these benefits. Moreover, synonymous keywords were combined using the “OR” logical operator. The first, second and third groups of keywords were then merged using the “AND” logical operator. EndNote software version 20.2.1 was used to manage the references.

**Table 1.** Search strategy utilized within the systematic review

Databases or sources	Cochrane Library, PubMed, ProQuest and Scopus.
Time–period	2000–2024
#1 – Effects	effect OR effects OR effectiveness OR outcome* OR advantage* OR result* OR consequence* OR impact OR impacts OR influence OR influences
#2 – mHealth	mHealth OR mobile health
#3 – Mental health	depress* OR anxi* OR anxiety OR mental health OR mental illness* OR behavioral health OR obsess* OR obsessive behavior* OR panic*
Final strategy	**#1 AND #2 AND #3**

### Inclusion and exclusion standards

This study included articles published in English between the years 2000 and 2024. Studies that did not cover a relevant topic, lacked a title or abstract describing the topic, or lacked a title, abstract, or full-text providing any data relevant to the topic were excluded from this study. Additionally, other types of publications such as short communications, letters to the editor and other irrelevant publications were removed from consideration.

### Screening and data retrieval

During the screening process, any duplicates were removed and the remaining articles were screened by their title and abstract. Articles that were not relevant to the research objective were discarded, while the full text of the remaining articles was read. The research objective, as previously described, was to gather existing data on the advantages of mHealth utilization in mental health services. Only studies that met the eligibility criteria were included in the final analysis. The entire process was carried out independently by each of the authors. In case of any conflict regarding the results of the procedure, the authors consulted with each other to complete the screening process.

Data from the final studies that aligned with the study objective were extracted independently by one of the authors and the process was independently monitored and reconducted by another researcher. The authors used Microsoft Office Excel 2016 to create a data extraction form for data collection. The form consisted of sections such as authors, title, year, journal, indexed databases, study type, disorder type, number of final articles and summary of results.

### Quality appraisal of final articles using the Critical Appraisal Skills Programme checklist

The validity, relevance, bias and applicability of research studies were assessed using the Critical Appraisal Skills Programme (CASP) appraisal checklist professionally designed to assess the quality of systematic reviews, which is designed to evaluate the quality of selected studies. The checklist served as a tool to help determine the overall risk of bias and quality of the research.

The CASP checklist for systematic reviews consists of 10 questions that assess articles based on various criteria, including the validity of the results, the quality of the study and the applicability of the results (Programme, 2018). A scoring system was employed in which each question was assigned a score of 2 for a “yes” response, 1 for “cannot tell,” and 0 for “no.” The maximum possible score was 16, which corresponded to three levels of quality: low, medium and high. Only articles with an average score of 10 or higher were included in our review. The total score for each study was calculated and presented in the results section in percentage. After screening and evaluating the quality of studies using the CASP checklist, only the final studies with the desirable quality scores were included in our review.

### Data analysis

The objective of this stage was to determine the key themes of advantages of mHealth utilization in mental health services. A thematic analysis was performed by the authors on the data collected from the texts of the manuscripts of the final papers obtained through the systematic review. In this process, the authors employed Boyatzis’s code development approach to conduct their analysis (Boyatzis, 1998).

The thematic analysis was performed to identify the data relevant to the topic of the study. During the process, the authors initially familiarized themselves with the data by collaboratively reviewing the benefits derived from the final studies, which were consolidated into a single sheet using Microsoft Excel 2016. Subsequently, they generated initial codes from the data. Finally, based on the research question, they categorized the data into a single table and generated subthemes. In order to enhance the validity and reliability of the results and minimize the risk of error or bias, the authors repeated the steps of the thematic analysis independently and consulted with each other to resolve any disagreements that arose during the process.

## Results

The results of the study are presented in three parts: systematic review, quality assessment of final studies and thematic analysis.

### Systematic review

**Figure 1.**
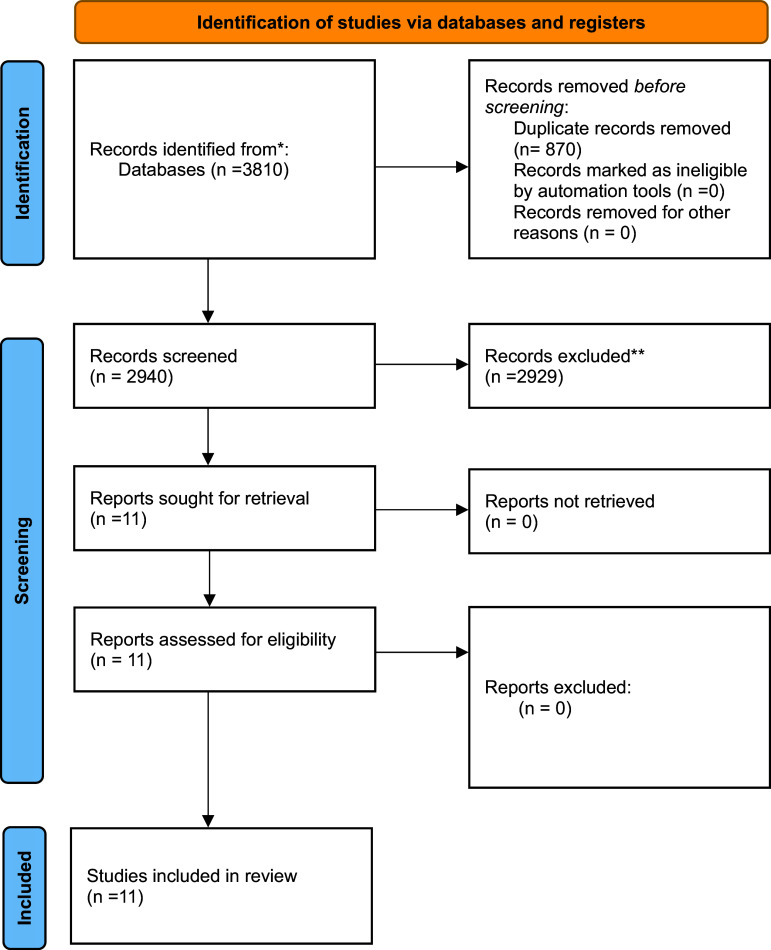
Preferred Reporting Items for Systematic Reviews and Meta-Analyses (PRISMA) diagram of the systematic review.

As shown in Figure 1, 3,810 studies were acquired by conducting a systematic search of relevant databases. Of these, 870 studies were identified as duplicates and excluded. After applying the inclusion and exclusion criteria, 11 papers were selected as final studies.

As delineated in Supplementary Appendix 1 (Bibliography of final studies); the final studies had the following characteristics:

- They were published in various medical journals between 2015 and 2022.
- They were indexed in databases such as PubMed, Scopus and Embase.
- They were all systematic reviews, except for three which were scoping reviews.
- They targeted various mental health conditions, such as psychiatric disorders, substance abuse disorders, self-harm, peripartum mood disorder, psychotic disorders, schizophrenia, schizoaffective disorder, psychosis, bipolar disorder, psychosocial health, depression, anxiety, sleep disorders, stress, panic disorders and postpartum depression.
- They included a range of 7–46 articles per study, with an average of 25 included articles per study.
- 36% of the final studies addressed the utilization of mHealth in low resource settings and vulnerable populations.

### Quality assessment of final studies

**Figure 2.**
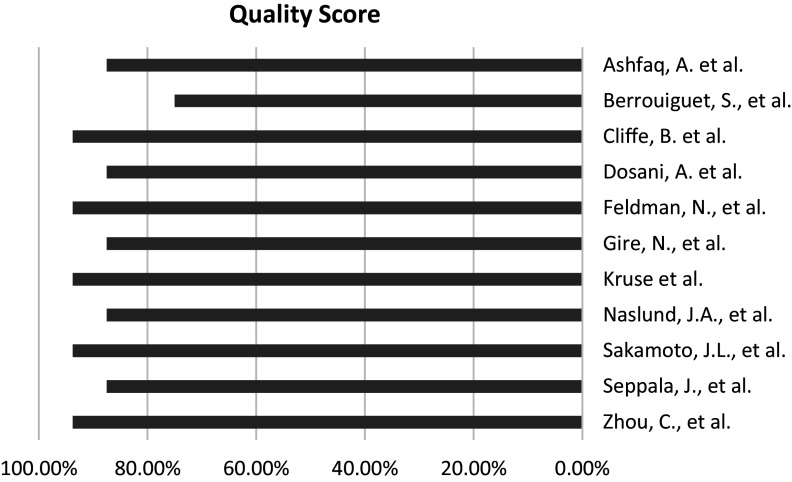
Summary of the findings of the quality assessment of the final studies.

As mentioned earlier, the CASP checklist was used to assess the validity of the results of 11 included studies. The studies were scored based on their answers to the CASP questions, with a maximum possible score of 16. As shown in Figure 2, the final scores ranged from 12/16 (75%) to 15/16 (93.75%), indicating that all of the studies were considered to be of good quality. The risk of bias within the final studies was shown to be in a convincing level. Furthermore, Supplementary Appendix 2 (Quality assessment of final studies) shows the full details of the quality assessment of the final studies.

The quality assessment showed that the authors of the final studies had addressed clearly focused questions and looked for the right type of papers. Moreover, they had included important and relevant studies and assessed their quality. The results seemed precise, but it was not always clear if they could be applied locally or if all important outcomes were considered. Furthermore, the advantages of conducting the studies in such contexts were considered to be worth the harms and costs.

### Thematic analysis

Table 2 presents the results of the thematic analysis on the final data. The findings highlighted the importance of incorporating mHealth interventions into mental health care strategies and suggested that mHealth interventions can improve: ‘Accessibility, Convenience and Adaptability’, ‘Patient-centeredness’, ‘Data insights’ and ‘Efficiency and Effectiveness’ in mental health services.

**Table 2.** Findings from the thematic analysis on the final data excerpted from the included studies

Theme	Subtheme	Reference
Accessibility, Convenience and Adaptability	Accessibility to low–resource settings and vulnerable groups	(Seppälä et al., 2019; Ashfaq et al., 2020; Cliffe et al., 2021; Kruse et al., 2022; Zhou et al., 2022)
	Increased access to evidence–based care	(Feldman et al., 2021)
	Convenience	(Cliffe et al., 2021)
	Adaptability	(Berrouiguet et al., 2016)
Patient–centeredness	Encouraging treatment in resistant populations	(Berrouiguet et al., 2016)
	Positive user experience	(Cliffe et al., 2021)
	Interactivity	(Berrouiguet et al., 2016; Feldman et al., 2021)
	Adherence	(Naslund et al., 2015)
	Patient empowerment	(Feldman et al., 2021)
	Personalization	(Naslund et al., 2015; Sakamoto et al., 2022; Zhou et al., 2022)
	Timeliness	(Seppälä et al., 2019)
Data insights	Real–time data	(Feldman et al., 2021)
	Objective data	(Seppälä et al., 2019)
	Long–term data	(Seppälä et al., 2019)
Efficiency and Effectiveness	Cost–saving	(Naslund et al., 2015; Kruse et al., 2022)
	Treatment outcomes	(Naslund et al., 2015; Gire et al., 2017; Ashfaq et al., 2020; Dosani et al., 2020; Cliffe et al., 2021; Feldman et al., 2021; Kruse et al., 2022; Sakamoto et al., 2022; Zhou et al., 2022)

Accessibility, Convenience and Adaptability: mHealth interventions can provide easy access to evidence-based care and can be accessed from anywhere and at any time. This makes them a convenient option for individuals who may not have the time or resources to attend face-to-face therapy sessions. Additionally, mHealth interventions can provide accessible and convenient support for vulnerable groups such as postpartum women with depression, especially in low- and middle-income countries. Furthermore, text messaging is highly adaptable to any health care strategy as it does not interfere with preexisting care procedures.

Patient-centeredness: mHealth interventions can be attractive to patients who may be resistant to traditional forms of mental health treatment. Text messaging can be used to reach patients with severe mental illness or those who live in remote areas. Additionally, some users have reported feeling more comfortable and open with their therapist when receiving virtual therapy compared to face-to-face therapy. mHealth interventions can also increase interactivity between patients and healthcare providers, improve adherence rates through medication reminders, empower patients to take control of their mental health and provide personalized care tailored to individual needs.

Data insights: mHealth interventions can provide real-time data through sensors embedded in smartphones. This data can provide valuable insights into patterns, trends and fluctuations in symptoms and behaviors over time. Additionally, mobile devices can collect objective data on physiological and mental states in a transparent and unobtrusive way.

Efficiency and Effectiveness: mHealth interventions can be cost-effective compared to traditional face-to-face therapy, making them a more accessible option for individuals with limited financial resources. Furthermore, some studies have reported reductions in symptoms of mental health difficulties such as anxiety, schizophrenia and depression through the use of mHealth interventions.

## Discussion

As the findings from the review delineated, almost half of the papers (36%) reported the advantages of the utilization of mHealth on mental health services. These papers referred to the positive outcomes of the intervention in low-resource settings and vulnerable populations, which presented the significant potential of mHealth utilization in enhancement of mental health services in such contexts (Ashfaq et al., 2020; Dosani et al., 2020; Feldman et al., 2021; Sakamoto et al., 2022).

As the findings from the quality assessment delineated, the mean score of the quality assessment of the included studies was 89.2%, presenting their convincing status in terms of levels of quality and risk of bias. This finding underscored the robustness and precision of the included studies, demonstrating their high degree of validity and reliability.

The findings from the thematic analysis of the data acquired from the final studies revealed that the advantages of mHealth utilization in mental health services were classified into four themes: ‘Accessibility, Convenience and Adaptability’, ‘Patient-centeredness’, ‘Data insights’ and ‘Efficiency and Effectiveness’. In this section of the study, we aimed to analyze and discuss each theme with the literature at hand.

### Accessibility, convenience and adaptability

Accessibility and convenience are major advantages of utilization of mHealth within mental health services: mHealth has demonstrated to provide a feasible and practical model of healthcare service delivery for mental health patients (Ashfaq et al., 2020; Cliffe et al., 2021; Zhou et al., 2022).

As the results of the study indicated, accessibility to vulnerable groups and the populations in low-resource settings and increased access to evidence-based care were significant advantages of utilization of mHealth in mental health services (Seppälä et al., 2019; Cliffe et al., 2021; Feldman et al., 2021; Zhou et al., 2022). Mobile phones can offer mental health support to many people who self-harm but do not seek help (Cliffe et al., 2021). Furthermore, mobile applications and telemedicine tools can also help postpartum women with depression, increasing accessibility to larger populations especially in low-resource settings where geographical distance and limited availability of mental health professionals pose considerable barriers (Seppälä et al., 2019; Zhou et al., 2022). Meanwhile, mHealth interventions can enhance the access to evidence-based care (Feldman et al., 2021).

Accessibility is demonstrated to be interconnected with several sub-domains: availability, utilization, affordability and acceptability. These interrelated sub-domains have significant roles in the evolution and implementation of the concept of accessibility (Gulliford et al., 2002). In such context, according to the findings of this study which are manifested in this theme and the other themes, mHealth interventions have demonstrated to satisfy all of these sub-domains and provide an available, utilizable, affordable and acceptable model of service delivery in mental health services (Naslund et al., 2015; Berrouiguet et al., 2016; Cliffe et al., 2021; Zhou et al., 2022).

On the other hand, a phenomenon has been examined recently within the literature of telehealth tools, which denotes the significant challenge of accessibility of telehealth tools to vulnerable populations with limitations in the access to the digital and internet-based tools (Ramsetty and Adams, 2020; Alkureishi et al., 2021; Saeed and Masters, 2021). Digital exclusion is the phenomenon of being deprived of the advantages that the digital world offers, which may affect those who experience the digital divide. It is shown as the provision of services via digital means increases at an unprecedented rate, the possibility of being excluded becomes more pronounced (Spanakis et al., 2021).

Despite ongoing efforts to equip under-resourced communities with technology for telehealth and online health information access, disparities in race, income, education and age hinder effective use of this technology for healthcare (Campbell et al., 2019). It is suggested that initiatives promoting digital inclusion, such as affordable broadband, internet-enabled devices, digital literacy training, technical support and self-sufficient, collaborative application design, are crucial to enhance mHealth access in these settings (Sieck et al., 2021).

The results of our study showed that mHealth interventions offer a convenient option for individuals who may face difficulties in attending face-to-face therapy sessions due to time or resource constraints, as they can be accessed from anywhere and anytime (Cliffe et al., 2021). As an example, it has been shown that the implementation of mHealth, which provides telehealth, could potentially be a significant advancement for Muslim women in certain regions of the world who have specific values and preferences. This is because Muslim women, as adherents of Islam, generally avoid unnecessary socialization, particularly with the opposite gender, due to their beliefs (Inhorn and Serour, 2011).

In terms of adaptability, text messaging is a highly versatile tool that can be seamlessly integrated into any healthcare strategy, as it does not disrupt preexisting care procedures (Berrouiguet et al., 2016); Therefore, the utilization of mHealth platforms by policymakers can be an attractive approach due to their capability to be implemented in diverse and volatile circumstances, taking into account the situational strengths and weaknesses.

### Patient-centeredness

As our findings showed, patient-centeredness as one of the core advantages of utilization of mHealth in mental health services can be categorized into several domains: attractiveness, interactivity, adherence, patient empowerment, personalization and timeliness (Naslund et al., 2015; Berrouiguet et al., 2016; Seppälä et al., 2019; Cliffe et al., 2021; Feldman et al., 2021; Sakamoto et al., 2022; Zhou et al., 2022). Patient-centeredness or the delivery of healthcare services that is centered around the patient has been associated with eight distinct principles. These principles include respect for the preferences of patients, coordination and integration of care, provision of information and education, ensuring physical comfort, providing emotional support, involving family and friends, ensuring continuity and transition and finally, ensuring access to care (America IoMUCoQoHCi, 2001). The concept of patient-centeredness encompasses various dimensions and components. By identifying these dimensions and components, it is possible to enhance the quality of healthcare services. This can be achieved through the development of measures to evaluate patient-centeredness and by providing feedback to healthcare providers (Cramm and Nieboer, 2013).

Encouraging treatment in resistant populations and positive user experience are major advantages of utilization of mHealth in mental health services: they generate an appealing atmosphere for patients to participate more in the services delivered by mHealth (Berrouiguet et al., 2016; Cliffe et al., 2021). In this context, text messaging can serve as a means to reach patients who may be resistant to traditional forms of mental health treatment. This includes individuals with severe mental illness or those residing in remote areas (Berrouiguet et al., 2016). Additionally, some users of mHealth interventions have reported feeling more comfortable and open with their therapist when receiving virtual therapy as opposed to face-to-face therapy (Cliffe et al., 2021).

The utilization of mHealth text messaging as a means of communication between patients and healthcare providers facilitates more frequent and instantaneous interactions. This can enhance patient engagement, enable more informed treatment decisions and ultimately lead to improved treatment outcomes (Berrouiguet et al., 2016; Feldman et al., 2021). Higher interactivity between patients and healthcare providers can play a significant role quality enhancement and robustness of the service delivery network, since, it has been demonstrated that several key elements contribute to patient-centeredness and the enhancement of service delivery quality, ultimately leading to greater patient satisfaction. These elements include providing patients with clear and understandable information about their condition, medications and potential side effects; verifying that patients comprehend the information provided; addressing any inquiries patients may have; informing patients about follow-up care; and using language that is easily understood by patients (Gorawara-Bhat and Cook, 2011; Hashim, 2017; Kwame and Petrucka, 2021).

Adherence can be defined as the extent to which a patient’s behavior aligns with the recommendations and medication prescriptions provided by their healthcare provider (Panahi et al., 2022). Adherence is known to be a crucial factor within the process of healthcare service delivery, since, any non-adherence by patients can have significant economic consequences, not only for the individual but also for the healthcare system (Mallow et al., 2014). Moreover, in cases of chronic illness, non-adherence to prescribed treatment may result in negative clinical consequences (Yap et al., 2016). The utilization of text messaging as a means of reminding patients to take their medication has been demonstrated to enhance adherence rates (Naslund et al., 2015). Hence, mHealth can positively impact the improvement of adherence among mental health patients. This makes the utilization of such platforms a strategic policy for healthcare policymakers to enhance the quality of care through simple approaches, such as improving patient adherence.

Mobile health interventions have the potential to empower individuals to take charge of their mental well-being by providing them with the necessary tools and resources to effectively manage their symptoms. This approach can facilitate a greater sense of control and autonomy for patients in managing their mental health (Sakamoto et al., 2022). It has been demonstrated that enabling patients to actively engage in their own healthcare, while also respecting their autonomy and preferences regarding treatment options, are essential components of a patient-centered approach (Madsen and Fraser, 2015; Kambhampati et al., 2016).

Digital health interventions can be customized to accommodate the unique requirements and inclinations of each individual suffering from serious mental illness (Naslund et al., 2015; Sakamoto et al., 2022; Zhou et al., 2022). Prioritizing user preferences can improve their perception of healthcare services and achieve patient-centeredness. Person-centered therapy, developed by Carl Rogers, is a nondirective approach that focuses on the client’s self-actualization and healing. In such process, the therapist’s attitude, relationship and empathy are considered crucial (Yao and Kabir, 2023).

By leveraging data obtained through sensors, mHealth can enable interventions to be delivered in a timely manner. The data collected can be analyzed to anticipate changes in clinical conditions and guide the provision of personalized interventions based on the specific needs of each individual (Seppälä et al., 2019). Numerous research studies have highlighted the importance of promptness and timeliness in the delivery of healthcare services as a crucial component of patient-centered care (Smart and Titus, 2011; Ng et al., 2021); Therefore, by incorporating mHealth technology into mental health services, it is possible to address the issue of timeliness, which is a fundamental component of patient-centered care. This can be achieved through the effective utilization of mHealth tools and resources.

### Data insights

One of the significant advantages of utilizing mHealth on mental health services is the availability of data insights. This is made possible through the ability of mHealth to produce real-time, objective and long-term data (Seppälä et al., 2019; Feldman et al., 2021). Smartphones are equipped with sensors that can collect data related to various aspects of an individual’s life, including activity, sleep and energy levels. This real-time data provides a unique opportunity to gain insight into perinatal mental health by allowing us to understand the impact of both environmental and temporal factors on an individual’s clinical state. This information can be used to improve our understanding and treatment of perinatal mental health conditions (Feldman et al., 2021). Furthermore, Mobile devices equipped with sensors have the capability to gather quantitative markers of behavior and functionality in a nonintrusive and seamless manner. This offers an objective measure of physiological and psychological states, supplementing self-reported data to provide a more holistic understanding of an individual’s condition (Seppälä et al., 2019).

Mobile devices facilitate the prolonged observation of individuals who have mental health conditions. This enables the acquisition of valuable insights regarding the patterns, trends and variations in symptoms and behaviors over time, which can be utilized to inform treatment planning and decision-making (Seppälä et al., 2019). Long-term datasets can reveal valuable insights and patterns that may not be discernible in shorter-term data. This can result in the development of more robust and effective machine learning models, particularly when utilizing these long-term datasets (Gianfrancesco et al., 2018). Machine learning techniques, including AI and big data analytics, have been utilized in mHealth to enhance healthcare systems and offer personalized insights (Istepanian and Al-Anzi, 2018).

While mHealth offers valuable data insights, it also presents challenges such as data privacy and security. The use of mHealth applications and smart speakers can lead to unintentional personal information disclosure due to active microphones and sensitive health data. The absence of security guidelines, developer expertise and secure data transmission are significant hurdles in creating secure mHealth apps. The intertwined nature of security and privacy concerns in mHealth apps handling patient data underscores the need for strong security measures (Aljedaani and Babar, 2021; Schroeder et al., 2022).

### Efficiency and effectiveness

Efficiency in healthcare pertains to the optimal utilization of resources to achieve desired outcomes. It involves comparing the outputs of a delivery system, such as physician visits, relative value units, or health outcomes, with the inputs, including cost, time and materials. This comparison allows for the determination of the most effective use of resources in achieving the desired outcomes (Allin et al., 2016; Mbau et al., 2023). Mobile health interventions have been demonstrated to be cost-effective in comparison to traditional face-to-face therapy. This may make them a more viable option for individuals with limited financial resources (Naslund et al., 2015).

In the context of healthcare, effectiveness refers to the capacity of a particular intervention to produce a significant impact on patients under routine clinical circumstances (Enrique and Marta, 2020). According to some studies, mHealth interventions have been shown to be effective in reducing symptoms of various mental health conditions, including anxiety, schizophrenia, depression and borderline personality disorder. These interventions have been particularly effective when used as standalone treatments (Naslund et al., 2015; Seppälä et al., 2019; Ashfaq et al., 2020; Dosani et al., 2020; Cliffe et al., 2021; Feldman et al., 2021; Sakamoto et al., 2022; Zhou et al., 2022).

## Limitations and implications

This study was not able compare the advantages of mHealth interventions in comparison to other forms of mental health treatment, such as face-to-face therapy or medication, due to the limited time and volume of the manuscript. Future research could investigate the comparative advantages of different treatment modalities. Furthermore, due to the nascent nature of the mHealth project and the limited number of reviews within the literature on the topic, the results of this study may lack the precision that could be obtained through long-term analysis of the advantages of utilizing mHealth platforms in mental health services. Due to the limited scope and volume of the paper, we could not investigate the existing challenges regarding mHealth utilization in mental health services in details; this can be an implication for future researchers to investigate such phenomenon. The study suggests that mHealth interventions can be a promising option for delivering mental health services, particularly in low-resource settings or during public health emergencies. Furthermore, the study calls for further research to explore the potential of mHealth utilization within mental health care, particularly in vulnerable populations and low-resource settings.

## Conclusions

This study conducted a systematic review of existing review papers on the advantages of mHealth utilization on mental health services. The study identified and categorized the main advantages of mHealth interventions into four major themes: ‘Accessibility, Convenience and Adaptability’, ‘Patient-centeredness’, ‘Data insights’ and ‘Efficiency and Effectiveness’. The findings suggested that mHealth interventions can be a viable and promising option for delivering mental health services to large and diverse populations particularly in vulnerable groups and low-resource settings.

## Supporting information

Khosravi and Azar supplementary material 1 Khosravi and Azar supplementary material

Khosravi and Azar supplementary material 2 Khosravi and Azar supplementary material

Khosravi and Azar supplementary material 3 Khosravi and Azar supplementary material

## Data Availability

The research data can be accessed by contacting the corresponding author.

## List of abbreviations

CASP: Critical Appraisal Skills Programme
mHealth: mobile health
PRISMA: Preferred Reporting Items for Systematic Reviews and Meta-Analyses

## Supplementary material

The supplementary material for this article can be found at http://doi.org/10.1017/gmh.2024.39.
