# Supplementary material for: A systematic review of reviews on the advantages of mHealth utilization in mental health services: A viable option for large populations in low-resource settings
Source: Glob Ment Health (Camb). 2024 Apr 4;11:e43. doi: 10.1017/gmh.2024.39 (PMC11058521; doi:10.1017/gmh.2024.39)
Supplement: Khosravi and Azar supplementary material 1 — Khosravi and Azar supplementary material [file S2054425124000396sup001.docx]

**Appendix 1. Bibliography of final studies.**

| No. | Year | Journal | Indexed databases | Study type | Targeted Disorder | Number of final articles | Reference |
| --- | --- | --- | --- | --- | --- | --- | --- |
| 1 | 2020 | international journal of environmental research and public health | PubMed, Scopus, Embase | Systematic review | Mental health disorders | 10 | (Ashfaq et al. 2020) |
| 2 | 2016 | Journal of Medical Internet Research (JMIR) | PubMed, Scopus, Embase | Systematic review | Psychiatric conditions and substance abuse disorders | 36 | (Berrouiguet et al. 2016) |
| 3 | 2021 | Journal of Medical Internet Research (JMIR) | PubMed, Scopus, Embase | Scoping review | self-harm | 36 | (Cliffe et al. 2021) |
| 4 | 2020 | international journal of environmental research and public health | PubMed, Scopus, Embase | Scoping review | Perinatal depression | 12 | (Dosani et al. 2020) |
| 5 | 2021 | Journal of Behavioral Medicine | PubMed, Scopus | Systematic review | peripartum mood disorder | 9 | (Feldman et al. 2021) |
| 6 | 2017 | mhealth | PubMed, Scopus | Systematic review | psychotic disorders | 7 | (Gire et al. 2017) |
| 7 | 2022 | Journal of Medical Internet Research (JMIR) | PubMed, Scopus, Embase | Systematic review | anxiety, sleep disturbance, anger, pain, fatigue, digestive disturbance, decision conflict, decision regret, and body image distress | 33 | (Kruse et al. 2022) |
| 8 | 2015 | Journal of mental health | PubMed, Scopus, Embase | Scoping review | schizophrenia, schizoaffective disorder, psychosis, or bipolar disorder | 46 | (Naslund et al. 2015) |
| 9 | 2022 | BMJ open | PubMed, Scopus, Embase | Systematic review | Psychosocial health | 44 | (Sakamoto et al. 2022) |
| 10 | 2018 | JMIR Mental Health | PubMed, Scopus | Systematic review | depression, anxiety, sleep disorders, psychotic disorders, stress, and panic disorders. | 35 | (Seppälä et al. 2019) |
| 11 | 2020 | Journal of Telemedicine and Telecare | PubMed, Scopus | Systematic review | postpartum depression (PPD) | 11 | (Zhou et al. 2022) |

**Ashfaq A, Esmaili S, Najjar M, Batool F, Mukatash T, Al-Ani HA and Koga PM** (2020) Utilization of Mobile Mental Health Services among Syrian Refugees and Other Vulnerable Arab Populations-A Systematic Review. *Int J Environ Res Public Health* **17**(4). <https://doi.org/10.3390/ijerph17041295>.

**Berrouiguet S, Baca-García E, Brandt S, Walter M and Courtet P** (2016) Fundamentals for Future Mobile-Health (mHealth): A Systematic Review of Mobile Phone and Web-Based Text Messaging in Mental Health. *J Med Internet Res* **18**(6)**,** e135. <https://doi.org/10.2196/jmir.5066>.

**Cliffe B, Tingley J, Greenhalgh I and Stallard P** (2021) mHealth Interventions for Self-Harm: Scoping Review. *J Med Internet Res* **23**(4)**,** e25140. <https://doi.org/10.2196/25140>.

**Dosani A, Arora H and Mazmudar S** (2020) mHealth and perinatal depression in low-and middle-income countries: A scoping review of the literature. *International Journal of Environmental Research and Public Health* **17**(20)**,** 1-18. <https://doi.org/10.3390/ijerph17207679>.

**Feldman N, Back D, Boland R and Torous J** (2021) A systematic review of mHealth application interventions for peripartum mood disorders: trends and evidence in academia and industry. *Arch Womens Ment Health* **24**(6)**,** 881-892. <https://doi.org/10.1007/s00737-021-01138-z>.

**Gire N, Farooq S, Naeem F, Duxbury J, McKeown M, Kundi PS, Chaudhry IB and Husain N** (2017) mHealth based interventions for the assessment and treatment of psychotic disorders: a systematic review. *Mhealth* **3,** 33. <https://doi.org/10.21037/mhealth.2017.07.03>.

**Kruse CS, Betancourt JA, Gonzales M, Dickerson K and Neer M** (2022) Leveraging Mobile Health to Manage Mental Health/Behavioral Health Disorders: Systematic Literature Review. *JMIR Ment Health* **9**(12)**,** e42301. <https://doi.org/10.2196/42301>.

**Naslund JA, Marsch LA, McHugo GJ and Bartels SJ** (2015) Emerging mHealth and eHealth interventions for serious mental illness: a review of the literature. *J Ment Health* **24**(5)**,** 321-332. <https://doi.org/10.3109/09638237.2015.1019054>.

**Sakamoto JL, Carandang RR, Kharel M, Shibanuma A, Yarotskaya E, Basargina M and Jimba M** (2022) Effects of mHealth on the psychosocial health of pregnant women and mothers: a systematic review. *BMJ Open* **12**(2). <https://doi.org/https://doi.org/10.1136/bmjopen-2021-056807>.

**Seppälä J, De Vita I, Jämsä T, Miettunen J, Isohanni M, Rubinstein K, Feldman Y, Grasa E, Corripio I, Berdun J, D'Amico E and Bulgheroni M** (2019) Mobile Phone and Wearable Sensor-Based mHealth Approaches for Psychiatric Disorders and Symptoms: Systematic Review. *JMIR Ment Health* **6**(2)**,** e9819. <https://doi.org/10.2196/mental.9819>.

**Zhou C, Hu H, Wang C, Zhu Z, Feng G, Xue J and Yang Z** (2022) The effectiveness of mHealth interventions on postpartum depression: A systematic review and meta-analysis. *J Telemed Telecare* **28**(2)**,** 83-95. <https://doi.org/10.1177/1357633x20917816>.
