# Supplementary material for: A systematic review of reviews on the advantages of mHealth utilization in mental health services: A viable option for large populations in low-resource settings
Source: Glob Ment Health (Camb). 2024 Apr 4;11:e43. doi: 10.1017/gmh.2024.39 (PMC11058521; doi:10.1017/gmh.2024.39)
Supplement: Khosravi and Azar supplementary material 2 — Khosravi and Azar supplementary material [file S2054425124000396sup002.docx]

**Appendix 2. Quality assessment of final studies included in the study utilizing the CASP checklist.**

| **Article No.** | **Are the results of the review valid?** | | **Is it worth continuing?** | | | **What are the results?** | | **Will the results help locally?** | | | **Final score** | **Reference** |
| --- | --- | --- | --- | --- | --- | --- | --- | --- | --- | --- | --- | --- |
|  | **Did the review address a clearly focused question?** | **Did the authors look for the right type of papers?** | **Do you think all the important, relevant studies were included?** | **Did the review’s authors do enough to assess quality of the included studies?** | **If the results of the review have been combined, was it reasonable to do so?** | **What are the overall results of the review?** | **How precise are the results?** | **Can the results be applied to  the local population?** | **Were all important outcomes considered?** | **Are the benefits worth the  harms and costs?** |  |  |
| 1 | Yes | Yes | Yes | No | Yes | The overall results of the review show positive impacts of mHealth on the access to services and on treatment outcomes among Syrian refugees. Despite the positive impacts, there are barriers to the uptake of mHealth applications. | The results seem to be precise. | Yes | Yes | Yes | **14/16**  **(87.5%)** | **(Ashfaq et al. 2020)** |
| 2 | Yes | Yes | Yes | No | Yes | The overall results of the review were that text messaging interventions have the potential to improve health outcomes in patients with psychiatric conditions and substance abuse disorders. The results were presented descriptively, and no odds ratios or other statistical measures were reported. | The results seem to be precise. | Cant tell | Cant tell | Yes | **12/16**  **(75%)** | **(Berrouiguet et al. 2016)** |
| 3 | Yes | Yes | Yes | Yes | Yes | The overall results of the review suggest that mHealth interventions may be effective in reducing self-harm behaviors and improving mental health outcomes. However, the authors note that the quality of the studies included in the review was variable, and that more research is needed to determine the effectiveness of specific mHealth interventions | The results seem to be precise. | Cant tell | Yes | Yes | **15/16**  **(93.75%)** | **(Cliffe et al. 2021)** |
| 4 | Yes | Yes | Yes | No | Yes | The overall results of the review demonstrated that depressive symptoms improved after the interventions delivered through mobile phones for the treatment of perinatal depressive symptoms in low- and middle-income countries. | The results seem to be precise. | Yes | Yes | Yes | **14/16**  **(87.5%)** | **(Dosani et al. 2020)** |
| 5 | Yes | Yes | Yes | Yes | Yes | The overall results of the review were that mHealth interventions have the potential to improve peripartum mood disorders, including depression and anxiety. The results were expressed in terms of the characteristics of the academic publications and mobile apps that were included in the review. The authors did not report any statistical analyses or effect sizes. | The results seem to be precise. | Cant tell | Yes | Yes | **15/16**  **(93.75%)** | **(Feldman et al. 2021)** |
| 6 | Yes | Yes | Cant tell | Yes | Yes | The overall results of the review suggest that mHealth interventions have the potential to improve access to mental health care, increase treatment adherence, and reduce costs, making them a promising tool for the assessment and treatment of mental health disorders | The results seem to be precise. | Cant tell | Yes | Yes | **14/16**  **(87.5%)** | **(Gire et al. 2017)** |
| 7 | Yes | Yes | Yes | Yes | Yes | The overall results of the review showed that mHealth interventions show promise in improving symptoms of ​depression, ​anxiety, quality of life, mental well-being, and other related outcomes. | The results seem to be precise. | Cant tell | Yes | Yes | **15/16**  **(93.75%)** | **(Kruse et al. 2022)** |
| 8 | Yes | Yes | Yes | Cant tell | Yes | The overall results of the review were that mHealth and eHealth interventions have the potential to improve outcomes for individuals with serious mental illness, particularly in the areas of self-management, medication adherence, psychoeducation, and symptom monitoring | The results seem to be precise. | Cant tell | Yes | Yes | **14/16**  **(87.5%)** | **(Naslund et al. 2015)** |
| 9 | Yes | Yes | Yes | Yes | Yes | The overall results of the review were that mHealth interventions have the potential to improve peripartum mood disorders, including depression and anxiety. The results were expressed in terms of the characteristics of the academic publications and mobile apps that were included in the review. The authors did not report any statistical analyses or effect sizes. | The results seem to be precise. | Cant tell | Yes | Yes | **15/16**  **(93.75%)** | **(Sakamoto et al. 2022)** |
| 10 | Yes | Yes | Cant tell | No | Yes | The overall results of the review suggest that mobile phone and wearable sensor-based mHealth approaches have the potential to improve mental health outcomes and symptoms. | The results seem to be precise. | Cant tell | Yes | Yes | **14/16**  **(87.5%)** | **(Seppälä et al. 2019)** |
| 11 | Yes | Yes | Yes | Yes | Yes | The overall results of the review showed that mHealth interventions were effective in reducing postpartum depression symptoms. The results were expressed as mean difference (MD) and standard error (SE) of continuous outcomes. | The results seem to be precise. | Cant tell | Yes | Yes | **15/16**  **(93.75%)** | **(Zhou et al. 2022)** |

***Legend 1 Critical Appraisal Skills Programme (CASP) questions scoring: Yes =2; Can’t Tell = 1; No = 0***

**Ashfaq A, Esmaili S, Najjar M, Batool F, Mukatash T, Al-Ani HA and Koga PM** (2020) Utilization of Mobile Mental Health Services among Syrian Refugees and Other Vulnerable Arab Populations-A Systematic Review. *Int J Environ Res Public Health* **17**(4). <https://doi.org/10.3390/ijerph17041295>.

**Berrouiguet S, Baca-García E, Brandt S, Walter M and Courtet P** (2016) Fundamentals for Future Mobile-Health (mHealth): A Systematic Review of Mobile Phone and Web-Based Text Messaging in Mental Health. *J Med Internet Res* **18**(6)**,** e135. <https://doi.org/10.2196/jmir.5066>.

**Cliffe B, Tingley J, Greenhalgh I and Stallard P** (2021) mHealth Interventions for Self-Harm: Scoping Review. *J Med Internet Res* **23**(4)**,** e25140. <https://doi.org/10.2196/25140>.

**Dosani A, Arora H and Mazmudar S** (2020) mHealth and perinatal depression in low-and middle-income countries: A scoping review of the literature. *International Journal of Environmental Research and Public Health* **17**(20)**,** 1-18. <https://doi.org/10.3390/ijerph17207679>.

**Feldman N, Back D, Boland R and Torous J** (2021) A systematic review of mHealth application interventions for peripartum mood disorders: trends and evidence in academia and industry. *Arch Womens Ment Health* **24**(6)**,** 881-892. <https://doi.org/10.1007/s00737-021-01138-z>.

**Gire N, Farooq S, Naeem F, Duxbury J, McKeown M, Kundi PS, Chaudhry IB and Husain N** (2017) mHealth based interventions for the assessment and treatment of psychotic disorders: a systematic review. *Mhealth* **3,** 33. <https://doi.org/10.21037/mhealth.2017.07.03>.

**Kruse CS, Betancourt JA, Gonzales M, Dickerson K and Neer M** (2022) Leveraging Mobile Health to Manage Mental Health/Behavioral Health Disorders: Systematic Literature Review. *JMIR Ment Health* **9**(12)**,** e42301. <https://doi.org/10.2196/42301>.

**Naslund JA, Marsch LA, McHugo GJ and Bartels SJ** (2015) Emerging mHealth and eHealth interventions for serious mental illness: a review of the literature. *J Ment Health* **24**(5)**,** 321-332. <https://doi.org/10.3109/09638237.2015.1019054>.

**Sakamoto JL, Carandang RR, Kharel M, Shibanuma A, Yarotskaya E, Basargina M and Jimba M** (2022) Effects of mHealth on the psychosocial health of pregnant women and mothers: a systematic review. *BMJ Open* **12**(2). <https://doi.org/https://doi.org/10.1136/bmjopen-2021-056807>.

**Seppälä J, De Vita I, Jämsä T, Miettunen J, Isohanni M, Rubinstein K, Feldman Y, Grasa E, Corripio I, Berdun J, D'Amico E and Bulgheroni M** (2019) Mobile Phone and Wearable Sensor-Based mHealth Approaches for Psychiatric Disorders and Symptoms: Systematic Review. *JMIR Ment Health* **6**(2)**,** e9819. <https://doi.org/10.2196/mental.9819>.

**Zhou C, Hu H, Wang C, Zhu Z, Feng G, Xue J and Yang Z** (2022) The effectiveness of mHealth interventions on postpartum depression: A systematic review and meta-analysis. *J Telemed Telecare* **28**(2)**,** 83-95. <https://doi.org/10.1177/1357633x20917816>.
