# Supplementary material for: A systematic review of reviews on the advantages of mHealth utilization in mental health services: A viable option for large populations in low-resource settings
Source: Glob Ment Health (Camb). 2024 Apr 4;11:e43. doi: 10.1017/gmh.2024.39 (PMC11058521; doi:10.1017/gmh.2024.39)
Supplement: Peer review history

# Editor report: Recommendation: A systematic review of reviews on the advantages of mhealth utilization in mental health services: a viable option for large populations in low-resource settings — R0/PR2

**DOI:** 10.1017/gmh.2024.39.pr2

**Affiliations:** Pravara Institute of Medical Sciences, India

No accompanying comment.

# Editor report: Decision: A systematic review of reviews on the advantages of mhealth utilization in mental health services: a viable option for large populations in low-resource settings — R0/PR3

**DOI:** 10.1017/gmh.2024.39.pr3

**Affiliations:** London School of Hygiene & Tropical Medicine, United Kingdom of Great Britain and Northern Ireland

No accompanying comment.

# Editor report: Recommendation: A systematic review of reviews on the advantages of mhealth utilization in mental health services: a viable option for large populations in low-resource settings — R1/PR5

**DOI:** 10.1017/gmh.2024.39.pr5

**Affiliations:** Pravara Institute of Medical Sciences, India

Thank you for addressing the reviewer comments. One reviewer has mentioned that there are a few typographical errors in the submission. Kindly revise and re-submit.

# Editor report: Decision: A systematic review of reviews on the advantages of mhealth utilization in mental health services: a viable option for large populations in low-resource settings — R1/PR6

**DOI:** 10.1017/gmh.2024.39.pr6

**Affiliations:** London School of Hygiene & Tropical Medicine, United Kingdom of Great Britain and Northern Ireland

No accompanying comment.
